# Types of laryngomalacia in children: interrelationship between clinical course and comorbid conditions

**DOI:** 10.1007/s00405-016-4334-5

**Published:** 2016-10-08

**Authors:** Beata Kusak, Ewa Cichocka-Jarosz, Urszula Jedynak-Wasowicz, Grzegorz Lis

**Affiliations:** 1 Department of Pediatrics, Chair of Pediatrics Jagiellonian University Medical College, Krakow, Poland; 2 University Children’s Hospital, Wielicka 265 str., 30-663 Krakow, Poland

**Keywords:** Laryngomalacia, Tracheomalacia, Bronchomalacia, Comorbidities

## Abstract

The aim of this study was to: (1) find out whether laryngomalacia (LM) types are related to clinical course; (2) which patients with LM are at higher risk of other airway malacia [tracheomalacia (TM) and/or bronchomalacia (BM)]; and (3) evaluate the prevalence of LM in our region. Patients with established LM diagnosis and complete clinical and endoscopy records were enrolled. They were classified into different LM types according to classification based on the side of supraglottic obstruction. One hundred ten children were included. The most common LM appearance was type I—58 children, followed by combine types (I + II and I + III)—38. The other airway malacia were found in 47 patients: TM in 31, BM in 10, and TM with BM in 6. Other comorbidities (cardiac, neurological, and genetic disorders) were identified in 30 children. Patients with combine types of LM differ from those with single type of LM in terms of prematurity (13 vs 31 %, *p* = 0.04) and higher weight on the examination day (*p* = 0.006). Patients with other airway malacia differ from children with isolated LM in terms of prematurity (40 vs 13 %, *p* = 0.008), comorbidities (38 vs 19 %, *p* = 0.024), and lower weight on the examination day (*p* = 0.014). The prevalence of clinically relevant LM was one in 2600–3100 newborns. Clinical course of LM cannot be anticipated on the basis of solely endoscopic evaluation of the larynx. Comorbidities and prematurity increase the risk of other airway malacia. The prevalence of LM is relatively high in the middle-south part of Poland.

## Introduction

Laryngomalacia (LM) is the most common laryngeal anomaly in infants [1]. The incidence of LM in the general population is unknown, but it is estimated to be around one in 2100–2600 children [2]. Its main but not only symptom is stridor [1]. LM can be associated with severe respiratory distress: respiratory failure, apnea, cyanosis, and feeding disorders: swallowing and feeding difficulties with choking, regurgitation, and microaspiration events [3, 4]. It is widely described that LM has an association with acid reflux, but there is no evidence for their causal relationship, and in fact, there is no widely approved recommendation for empiric antireflux treatment in those patients [5]. As a whole, it seems to be more important if infants with LM are developing well with appropriate weight gain. Due to increase work load while breathing and at the same time greater energy expenditure, LM may lead to failure to thrive.

Laryngomalacia is generally classified by its laryngoscopic appearance. The most commonly used classification, proposed by Olney et al. [6], is as follows: type 1—prolapse of the mucosa overlying the arytenoid cartilages, type 2—foreshortened aryepiglottic folds, and type 3—posterior displacement of the epiglottis. Various combinations of these types may be seen. Furthermore, LM can be accompanied by synchronous airway lesions (SAL), predominantly: other airway malacia [tracheomalacia (TM) and/or bronchomalacia (BM)], subglottic stenosis, and vocal cord paralysis. It has been shown that SAL are more frequent in infants with severe LM [1, 6–9].

The objective of this study was to investigate whether fiberobronchoscopic LM appearance (different types) relates to clinical status of the child and which patients with LM are at higher risk of other airway malacia. Secondary purpose of the study was to evaluate the prevalence of LM and concordance of other airway malacia with LM in our region.

## Patients and methods

The study was approved by the Jagiellonian University Bioethical Board (Protocol 122.6120.49.2015). Data were collected retrospectively. The fiberobronchoscopic (FB) database of the tertiary-level hospital (Department of Pulmonology, University Children’s Hospital in Krakow, Poland) was used to search for patients diagnosed with LM. There were following inclusion criteria in this study: (1) initial visit between January 2005 and December 2014; (2) diagnosis of LM established by the age of 24 months; (3) complete clinical and endoscopic patient records; and (4) complete history of perinatal period. A sum of 1264 FB examinations was reviewed and there were 138 patients identified with LM. One hundred and ten patients (62 boys, 56 %) fulfilled all the study inclusion criteria. The rest of the patients (*n* = 28) were excluded because of: incomplete records (*n* = 23) or age over 24 months on the day of examination (*n* = 5).

Fiberobronchoscopies were performed under topical anesthesia (lidocaine) and conscious sedation (midazolam). Laryngeal evaluations were done before lidocaine administration.

The following clinical and FB data were analyzed: (1) age at the time of diagnosis; (2) gestational age; (3) birth weight; (4) weight on the examination day; (5) main signs and symptoms related to LM; (6) comorbidities, i.e., genetic, cardiac, and neurological disorders; (7) types of LM according to classification proposed by Olney with authors’ modification as follows: type I—prolapse of the mucosa overlying the arytenoid cartilages or collapse of bulky arytenoid cartilages; type II—foreshortened aryepiglottic folds with the epiglottis curling; and type III—posterior displacement of the epiglottis; and (8) other airway malacia lesions (TM and/or BM).

Statistical analysis was conducted with the STATISTICA data analysis software system (version 10. StatSoft.Inc. http://www.statsoft.com). Statistical significance level was determined with *p* value of 0.05 or less. The birth weight and weight on the examination day were defined as percentile and identified by means of WHO charts (http://www.infantchart.com). For qualitative data, the Chi-square test was used and presented as odds ratio. Nonparametric tests (Wilcoxon test and *U* Mann–Whitney test) were used for quantitative data (age, percentile of birth weight, and percentile of weight on the examination day) and presented as median and interquartile rang (IQR).

The estimation of the prevalence of LM was calculated using officially reported numbers of live births in our region (Malopolskie voivodeship) (http://www.demografia.stat.gov.pl) and with the assumption that almost all the children with clinically relevant LM were referred to our hospital (the one and only tertiary-level hospital in the voivodeship).

## Results

The mean age of the studied children (*n* = 110) was 3.3 months (ranged 0.1–20.9 months) on the examination day. There were 27 (25 %) preterm infants with gestational age ranging from between 24 and 36 weeks of pregnancy. The following respiratory findings were observed: stridor in 82 children (75 %), respiratory distress in 26 (24 %), apnea in 18 (16 %), and protracted pneumonia due to aspiration events in 14 (13 %). Moreover, there were also observed feeding difficulties with choking in 20 children (18 %), but without any clinical relevant signs of gastro-esophageal reflux.

In 30 (27 %) children, other comorbidities were identified: congenital cardiac and/or vessels diseases (*n* = 14), inherited or perinatally acquired neurological diseases (*n* = 12), genetic disorders (*n* = 16), and others (for details, see Table 1).

**Table 1:** Feature of children with LM and comorbid conditions

Patient	Age (months)	Gender	Other comorbidity/comorbidities	Tracheostomy
1. PI	0.5	F	Vascular ring, dysmorphy	
2. GJ	0.7	F	Vascular ring	
3. SK	9.6	F	Vascular ring	
4. IJ	14.4	M	TOF	
5. GT	2.6	M	TOF, Smith–Magenis syndrome	
6. HM	5.8	M	IAA, ASD, VSD, diGeorge syndrome	
7. DM	2.4	M	PDA, PFO, MR	
8. NZ	1.9	F	ASD, cri du chat syndrome, corpus callosum hypoplasia	
9. BM	0.7	F	ASD, Arnold–Chiari syndrome, chromosomal aberration	
10. GB	4.8	M	CAVC, CHARGE syndrome	
11. PP	0.9	F	CAVC, Down syndrome	
12. NZ	3.8	F	CAVC, Down syndrome	
13. KA	6.1	M	CAVC, Down syndrome	
14. MA	2.1	F	ASD, Down syndrome	
15. RP	5.5	F	Down syndrome	
16. KK	1.2	M	Down syndrome	
17. JD	10.2	M	Down syndrome	
18. JM	11.1	M	Down syndrome	
19. ZA	0.3	F	CHARGE syndrome	
20. MA	2.1	M	Chromosomal aberration	
21. WK	0.6	M	Artogryposis	Tracheostomy
22. SP	0.5	F	Artogryposis	Tracheostomy
23. SM	1.4	F	Artogryposis	Tracheostomy
24. ZD	5.0	M	Corpus callosum dysgenesis	
25. BK	0.1	F	Corpus callosum agenesis	
26. CN	3.5	M	Myelomeningocele, Arnold–Chiari syndrome	Tracheostomy
27. KN	0.9	F	Encephalopathy	
28. TA	3.0	F	Encephalopathy	
29. GL	3.2	F	Encephalopathy	
30. KK	14.3	F	Encephalopathy	

*TOF* tetralogy of Fallot, *IAA* interrupted aortic arch, *ASD* atrial septal defect, *VSD* ventricular septal defect, *PDA* patent ductus arteriosus, *PFO* patent foramen ovale, *MR* mitral regurgitation, *CAVC* complete atrio-ventricular canal

As many as 88 (80 %) children demonstrated insufficient weight gain from birth. The median weight percentile on the examination day was significantly lower than the median birth weight percentile [20.85 (IQR: 3.30–47.40) vs 42.5 (IQR: 22.70–66.60)] (*p* = 0.0001), indicating discordant growth pattern in the observed children.

Detailed endoscopy revealed the following LM types: type I—in 58 children (53 %), type II—in 6 (5 %), type III—in 8 (7 %), and combine types—38 (35 %), i.e.: type I + II—in 29, type I + III—in 9 (there was no type II + III) among study children. The other airway malacia was found in 47 (43 %) patients: TM in 31 (28 %), BM in 10 (9 %), and TBM in 6 (6 %).

Clinical data revealed different patterns of associations with isolated LM or LM with other airway malacia (TM and/or BM). In group of LM with other airway malacia, there were predominantly preterm infants (*p* = 0.008), more children with comorbidities (*p* = 0.024), and lower weight on the examination day (*p* = 0.014) in comparison with children with isolated LM. However, there were no differences in birth weight and gender between these two groups (Table 2).

**Table 2:** Clinical comparisons between patients with isolated LM and with other airways malacia

	LM		OR (95 % CI)	*p* value
	Isolated *n* = 63	With: TM and/or BM *n* = 47		
Boys	36 (57 %)	26 (55 %)	0.93^a^ (0.43–1.99)	0.84
Preterm infants	8 (13 %)	19 (40 %)	4.67^b^ (1.82–11.98)	0.008
Comorbidities	12 (19 %)	18 (38 %)	2.64^c^ (1.12–6.24)	0.024
Age (months)	1.9 (0.70–3.50)	2.3 (0.10–5.60)	–	0.25
Birth weight (percentile)	42.9 (22.85–73.57)	42.1 (21.65–59.70)	–	0.32
Weight on examination day (percentile)	28.2 (5.70–51.30)	11.5 (0.50–35.85)	–	0.014

Parametric values: median and (interquartile rage)

*LM* laryngomalacia, *TM* tracheomalacia, *BM* bronchomalacia, *OR* odds ratio, *95* *% CI* (95 % confidence interval)

^a^Reference group = girls

^b^Reference group = term infants

^c^Reference group = children without comorbidities

Moreover, there were differences between children with single type of LM (types I or II or III) and those with combine types of LM (type I + II and type I + III). In the latter group, there were: significantly less preterm infants (*p* = 0.04), and significantly higher weight on the examination day as compared to group with single type of LM (*p* = 0.006). However, there were no such differences in the birth weight and gender (Table 3).

**Table 3:** Comparison between patients with single type of LM (types I or II or III) and patients with combine types of LM (type I + II and type I + III)

	LM		OR (95 % CI)	*p* value
	Single type *n* = 72	Combine types *n* = 38		
Boys	39 (54 %)	23 (61 %)	1.2^a^ (0.5–2.8)	0.52
Preterm infants	22 (31 %)	5 (13 %)	0.34^b^ (0.12–0.99)	0.04
Comorbidities	23 (32 %)	7 (18 %)	0.48^c^ (0.18–1.25)	0.088
Other airway malacia (i.e. LM with: TM and/or BM)	35 (49 %)	12 (32 %)	0.47^d^ (0.2–1.07)	0.06
Age (months)	2.15 (1.05–4.35)	1.65 (0.50–3.20)	–	0.16
Birth weight (percentile)	38.7 (18.95–63.15)	53.8 (29.50–71.60)	–	0.19
Weight on examination day (percentile)	11.9 (1.60–40.30)	34.95 (15.10–51.60)	–	0.006

Parametric values: median and (interquartile rage)

*LM* laryngomalacia, *TM* tracheomalacia, *BM* bronchomalacia, *OR* odds ratio, *95* *% CI* (95 % confidence interval)

^a^Reference group = girls

^b^Reference group = term infants

^c^Reference group = children without comorbidity

^d^Reference group = isolated LM

In the studied group of children, there were some interrelationships identified between LM, coexistence of other airway malacia, and general clinical conditions. As shown in Table 4, preterm babies with LM had significantly higher risk to have other airway malacia as compared to full-term babies with LM (*p* = 0.008). Moreover, children with comorbidities had higher chance of having other airway malacia compared to children without clinical disorders (*p* = 0.024). Gender was not the risk factor of other airway malacia in children with LM.

**Table 4:** Clinical risks of the other airway malacia (TM and/or BM) in the study children

	LM with: TM and/or BM *n* = 47	LM isolated *n* = 63	OR (95 % CI)	*p* value
Preterm infants	19 (40 %)	8 (13 %)	4.67^a^ (1.82–11.98)	0.0008
Comorbidities	18 (38 %)	12 (19 %)	2.64^b^ (1.12–6.24)	0.025

*LM* laryngomalacia, *TM* tracheomalacia, *BM* bronchomalacia, *OR* odds ratio, *95* *% CI* (95 % confidence interval)

^a^Reference group = full-term infants

^b^Reference group = infants without comorbidity

Because of incomplete records, it was not possible to characterize the group of excluded patients in detail. However, the excluded group did not differ from the study group in terms of gender [16 (57 %) boys, *p* = 0.94] and coexistence of other airway malacia [9 (56 %), *p* = 0.74], but mean age was 15.5 months (range 0.4–109.9 months) and was significantly higher (*p* = 0.005) than in the study group.

Analysis of data records of study group revealed that seven patients required surgical intervention. In all that cases, tracheostomy was done (median age of surgery 2 months, ranging from 0.5 to 5 months). All these children except one had either other comorbidities (see Table 1) or other airway malacia lesions (TM and/or BM). One patient with isolated LM (type I + III) needed early tracheostomy placement at the age of 2 weeks as a preventive management. He had lymphatic malformation in the neck and mouth area. All those patients demonstrated severe respiratory distress with apnea. The remaining patients (*n* = 103) did not require surgical intervention at the time of data collection (observation time from 1 to 11 years).

Out of 138 children with LM diagnosis, 113 were born in Malopolskie voivodeship. There were 349 754 live births during ten analyzed years in this voivodeship. The prevalence of LM calculated from that data was 1: 3100. Moreover, when the calculation of LM prevalence was restricted to data only from Krakow (capital city of voivodeship), it gave an estimation of 1: 2600 (28 LM patients out of 72,786 live births). Similar analysis showed prevalence of combine LM with TM and/or BM in Malopolskie voivodeship and Krakow, respectively, 1: 6700 and 1: 6600.

## Discussion

In general, LM is mild and self-limiting. For this reason, the majority of children with LM can be supervised by primary care physicians and there is no need for referral to laryngologist or pulmonologist. As a result, there is poor prevalence data of LM in the general population. Probably, mild and transient nature of LM contributes to some extent to the underestimation of this problem.

Most authors refer to Boogaard et al. estimation of primary airway malacia from the year 2005 [2]. We tried to estimate the prevalence of LM in the region (voivodeship), where our hospital functions as a referral center. The prevalence of clinically relevant LM was one in 2600–3100 newborns and was even higher when calculated separately for the main city (Krakow) population. Higher LM prevalence within the city boundaries emerged presumably from easier and wider accessibility to specialize medical care as well as higher awareness of parents and their concern of disease symptoms in their children.

As LM is not a rare disease, every pediatrician should be aware of the clinical needs of a child with this disorder. According to our results, almost 80 % of children with LM are at a significant risk of insufficient weight gain. This insufficient weight gain is likely caused by multiple features. There are some well-recognized reasons: increased effort of breathing, gastro-esophageal or laryngopharyngeal reflux disease, and in more severe cases, uncoordinated suck-swallow-breath sequence. On one hand, children with LM have higher caloric demands, and on the other hand, they may have feeding difficulties. Considering those facts, further studies should be directed towards finding out interventions which could improve weight gain (e.g., high calory formula intake, oro-facial rehabilitation, and surgical intervention).

Moreover, there is still uncertainty regarding the different forms of diagnostic approach to children with LM. Under debate is whether all infants with LM should undergo the complete airway examination (fiberobronchoscopy) or laryngeal evaluation only (laryngoscopy) [6–14]. Rates of SAL in children with LM range from 7.5 to 64 % [6–14] and strongly depend on patients’ population (higher incidence in those recruited at tertiary referral centers). The most common SAL feature is the other airway malacia (TM and/or BM) (ranging from 2 to 48 % [6–14]), followed by subglottic stenosis and vocal cord paralysis. The clinical significance of SAL is unclear. The need to perform fiberobronchoscopy instead of laryngoscopy as a method to evaluate SAL is a matter of discussion among experts. Some authors [7, 9, 11–13] accept the advantage of FB, while others [6, 10] suggest that patients with SAL will manifest the severity of this condition with clinical symptoms, urging the clinicians to perform complete airway examination anyway [6, 8].

Our study showed that there are two groups of patient with LM who are at a significantly higher risk of having TM and/or BM, namely, premature infants and children with accompanying diseases: congenital cardiac and/or vessels diseases, neurological diseases, and genetic disorders.

Recent studies have shown that patients with LM and other comorbidities have higher risk of negative outcome after supraglottoplasty and, therefore, are at risk of tracheostomy [15, 16]. Authors presume that it is related to the comorbidities: general hypotonia in children with neurological diseases [15], increased work of breathing in children with cardiac diseases [16], and anatomic airway obstruction in children with micrognathia [16]. However, our results lead us to the speculation that the negative outcome after supraglottoplasty is probably due to the coexistence of SAL.

Are there any other patterns that could decide on the clinical outcome of children with LM? Olney et al. proposed classification of LM that is simple and correlates with the surgical procedure if the patient requires a supraglottoplasty [6]. Van der Heijden et al. proposed a new classification system [Groningen Laryngomalacia Classification System (GLCS)] based on dynamic laryngeal changes [17], which also provide the type of surgical intervention. For the purpose of our study, to allocate all our patients, we modified Olney’s classification. In our opinion, it was similar to the GLCS. We wondered if the classification of LM was related to the other airway malacia and the clinical severity of disorders. Still, the LM etiology remains elusive. The best explanation of laryngeal malfunction gives the neurological theory [1, 3, 14]. These include delay in the development of neurological function and tone of laryngeal tissue which can lead to immature neuromuscular control of the airway patency. Theoretically, it can be assumed that combine types of LM are more immature and extensive and could be accompanied by the malacia of lower airways. It could increase the level of airway obstruction and, therefore, cause more severe symptoms. Such assumption has not been confirmed in our study. It has shown that children with combine types of LM had less frequent other airway malacia as compared to children with single types of LM and they were even better adapted to airway obstruction or the obstruction was less intensive which was reflected into better weight gain.

Could our results be extended to general population? In our study, there were many preterm infants (25 %) and infants with comorbidities (27 %). The percentage of children with the other airway malacia was also rather high (43 %). That is why the population of children enrolled in our study differs in some aspects from the most common book’s description of infants with congenital stridor (i.e., isolated LM) [1]. Those differences resulted probably from certain features of the hospital, where the study took place.

The study was performed in a tertiary-level hospital which has many specialized wards, i.e., neonatal intensive care unit and cardiac surgery unit. Endoscopic examinations of airway have been carried out by pulmonologists, and a routine complete airway evaluation (fiberobronchoscopy) is done under topical anesthesia and conscious sedation. Such circumstances enabled us to identify TM and BM (these could not be seen if a child is under general anesthesia and assisted ventilation [18]). Laryngeal evaluations were always done before administration of topical anesthesia.

We are aware that our population of children with LM is highly selected and, therefore, is not entirely representative of the general population of children with LM. However, it gives an insight into patients who should probably be referred to pulmonology centers.

## Conclusion

Clinical course of LM cannot be anticipated solely on the basis of endoscopic evaluation of the larynx. Comorbidities and prematurity increase the risk of the other airway malacia (TM and/or BM) and, therefore, can be a cause of negative outcome after supraglottoplasty. The vast majority of children with clinically relevant LM are at risk of insufficient weight gain.
